# Pediatric pain physician workforce: an assessment of supply and demand

**DOI:** 10.3389/fpain.2024.1390736

**Published:** 2024-10-15

**Authors:** Christopher B. Theriault, Tracy Burns, Kenneth Goldschneider, Anjali Koka, Deirdre Logan, Steven J. Weisman, Robert T. Wilder, R. Blake Windsor, William T. Zempsky

**Affiliations:** ^1^ Division of Pain and Palliative Medicine, Connecticut Children’s, Hartford, CT, United States; ^2^ Anesthesiology, Pain and Perioperative Medicine, Children’s National Hospital, Washington, DC, United States; ^3^ Pain Management Center, Cincinnati Children’s Hospital Medical Center, Cincinnati, OH, United States; ^4^ Department of Anesthesia, University of Cincinnati College of Medicine, Cincinnati, OH, United States; ^5^ Department of Anesthesiology, Critical Care and Pain Medicine, Boston Children’s Hospital, Boston, MA, United States; ^6^ Department of Psychiatry, Harvard Medical School, Boston, MA, United States; ^7^ Jane B. Pettit Pain and Headache Center, Children’s Wisconsin, Milwaukee, WI, United States; ^8^ Departments of Anesthesiology and Pediatrics, The Medical College of Wisconsin, Milwaukee, WI, United States; ^9^ Department of Anesthesiology and Perioperative Medicine, Mayo Clinic, Rochester, MN, United States; ^10^ Division of Pediatric Pain Medicine, Prisma Health Children’s Hospital, Greenville, SC, United States; ^11^ Department of Anesthesiology & Psychiatry, University of South Carolina School of Medicine, Columbia, SC, United States; ^12^ Department of Pediatrics, University of Connecticut School of Medicine, Farmington, CT, United States

**Keywords:** pediatrics, pain medicine, workforce shortage, training, access, chronic pain

## Abstract

**Introduction:**

Many youth with pain lack access to pediatric pain expertise. There is a critical shortage of pediatric pain physicians, due partly to a paucity of training programs in Pediatric Pain Medicine. Pain fellowships are Anesthesiology-based and there is no pathway to fellowship training or Pain Medicine board certification for pediatricians. This workforce assessment sought to examine the current state of Pediatric Pain Medicine in the United States and future interest in pursuing Pain Medicine among pediatricians.

**Methods:**

A multidisciplinary working group of Pain Medicine clinicians designed three surveys to examine pediatric comprehensive pain programs in the US, practice patterns of pediatricians and their motivations and opinions regarding Pain Medicine board certification, and current residents’ exposure to and interest in Pediatric Pain Medicine.

**Results:**

Wait times for initial evaluations are two months or longer for two-thirds of responding centers, and barriers to increase staff size and resources were reported, including an inadequate number of trained or available physicians. Pediatricians expressed interest in earning board certification in Pain Medicine, given the opportunity. Additionally, there is interest among pediatric residents in pursuing Pediatric Pain Medicine, and qualitative data highlight information residents perceived needing in order to pursue a career in the field.

**Discussion:**

Results demonstrate the need for increased training opportunities in pediatric pain medicine. A formal pathway to Pain Medicine for Pediatricians should be developed to increase the potential workforce and to address the lack of trained pediatric pain specialists, thereby improving access to care for youth with pain.

## Introduction

Pain is a common problem in children, whether acute (1), or chronic (2). Pain Medicine physicians are often needed as consultants for hospitalized children and for children and young adults who have chronic pain (3). Pediatric chronic pain prevalence rates vary greatly by pain location and diagnosis, but generally impact 15%–25% of children and adolescents (4). Pediatric pain conditions cause significant morbidity (4) and are associated with incremental health care costs of $1,339 per capita, and $11.8 billion total in the United States (US) (5). Unfortunately, there is a critical shortage of pediatric pain physicians, which reduces access leading to longer wait times and having to travel longer distances to receive treatment (6, 7) and leads to reduced health-related quality of life (8). Presently, only approximately half of the states in the US have a comprehensive pain program, primarily located in densely populated urban regions (9). Waitlist times for initial evaluations at an interdisciplinary pain clinic averaged 6.5 months, which was associated with anticipatory anxiety and frustration while pain and its functional impact went largely unchanged (7). A major factor contributing to the shortage is the lack of training programs in pediatric pain medicine (10). There are only eight accredited fellowship programs in the US in Pediatric Pain Medicine by the Accreditation Council for Graduate Medical Education (ACGME) (11). These are open to Anesthesiologists, Physiatrists, Psychiatrists and Neurologists, but, importantly, not Pediatricians (11).

In 2018, we created a panel made up of experts from multiple disciplines (Pediatric Pain Medicine, Pediatric Anesthesia, Anesthesia, Pediatrics, Psychology), across the field of Pediatric Pain Medicine to examine the current landscape of pediatric pain clinical care and to develop a plan to address workforce issues. All experts were nationally or internationally recognized with a history of publication, presentations and/or advocacy in the field. More specifically, we planned to evaluate the current state of the Pediatric Pain Medicine workforce, capture perceived barriers to care, understand practicing pediatrician’s perspectives on Pain Medicine board certification, and learn about the current level of capability and interest in Pain Medicine among pediatric residents.

## Materials and methods

Three surveys were designed for the purposes of this study. The first survey was distributed to 47 pediatric comprehensive pain programs in the US that were identified from the International Association for the Study of Pain website (9). A REDCap survey was sent via email to one identified leader within each program. The survey was sent a second time to those who did not respond initially. The survey included questions regarding program size, types of patients seen, number of physicians, training background of physicians, other services offered (i.e., acute pain, interventional, headache), and perceived barriers.

The second survey was designed to examine the current motivations and opinions regarding Pain Medicine board certification, as well as the current practice patterns of pediatricians currently practicing Pain Medicine in the US. A 10-question REDCap survey was sent to email listservs of Pediatric Pain, Pediatric Rheumatology, Pediatric Hospitalist Medicine, Pediatric Emergency Medicine, and Pediatric Palliative Medicine groups. The survey was sent a second time about four weeks later. The survey included questions regarding board certifications and sub-board certifications, interests and motivations in becoming board certified in Pediatric Pain Medicine, and professional time spent in various aspects of Pediatric Pain Medicine (i.e., acute pain management, chronic pain management, interventional pain management).

The third survey examined pediatric residents’ exposure and capability in Pain Medicine during their training, as well as their interest in a career in Pediatric Pain Medicine. A 27-question REDCap survey was sent to all pediatric residents with membership in the American Academy of Pediatrics (*n* = 10,000). The survey was sent a second time about four weeks later. The survey included questions regarding their current residency and exposure to Pain Medicine, capabilities in practicing Pediatric Pain Medicine (e.g., managing post-operative pain, performing trigger point injections), and interests in Pain Medicine fellowships or careers.

Descriptive statistics were used to summarize workforce survey results. For each survey, responses were characterized with frequencies and percentages for categorical variables, or means and standard deviations for continuous variables. Qualitative responses were grouped into general themes and reported with direct quotes from respondents.

## Results

### Survey 1

Thirty-four of the 47 (72%) identified programs responded. See Table 1 for a summary of the results.

**Table 1:** Descriptive statistics of survey 1 results.

	Programs	Percent
Pediatric Pain Mgmt Services	34	100%
Outpatient chronic pain	32	94%
Palliative care program	28	82%
Acute inpatient care	26	77%
Interventional therapies	26	77%
Headache clinic	20	58%
Pain rehab day program	9	27%
Physician FTE^a^	34	100%
<1	2	6%
1	11	32%
2–3	11	32%
4–5	2	6%
6–7	3	9%
8+	5	15%
Weekly New Patient Visits	32	100%
<5	14	44%
5–10	14	44%
>10	4	12%
Average Wait Times	34	100%
No wait	3	9%
1 month	8	24%
2 months	10	29%
3 months	8	24%
4 months	4	12%
5 months	0	0%
6 months	1	3%

^a^ 18 programs (52.9%) had at least one FTE physician certified in pain medicine.

#### Pediatric pain management services

The surveyed institutions provided a variety of pediatric pain management services including acute inpatient care (26 programs, 77%), outpatient chronic pain clinic (32 programs, 94%), pain rehabilitation day program (9 programs, 27%), pain rehabilitation inpatient program (12 programs, 35%), palliative care program (28 programs, 82%), interventional therapies (nerve blocks, etc.) (26 programs, 77%), and headache clinics (separate from pain clinic) (20 programs, 59%). One institution had no pain management services currently, but had one in the past.

#### Number of type of providers

Physician full-time equivalent (FTE) per program ranged from less than one (2 programs) to >8 (5 programs) with a median of 2 FTE. The number of mid-level providers per program ranged from less than one (9 programs) to >8 (3 programs). 18 programs (53%) had at least one FTE physician certified in Pain Medicine.

#### Training and certifications

The surveyed physicians report a variety of trainings and certifications that apply to their program: Anesthesiology (22, 65%), Pediatric Anesthesiology (25, 74%), Pediatrics (24, 71%), Pain Medicine (19, 56%), Physical Medicine and Rehabilitation (11, 32%), Neurology (5, 15%).

#### Waiting periods and new patient data

The waiting periods for each institution varied from no wait period to six months. 91% of the institutions had a wait period of 1 month or longer. The wait periods were as follows: no wait period (3 programs, 9%), 1 month (8 programs, 24%), 2 months (10 programs, 29%), 3 months (8 programs, 24%), 4 months (4 programs, 12%), 6 months (1 program, 3%). The number of new patients seen per week were as follows: under 5 (14, 44%), 5–10 (14, 44%), 11+ (4, 12%).

#### Barriers

26 programs (77%) surveyed thought that patient access and availability of services would benefit from increased physician FTE, while eight programs (24%) reported it would not. Of those that responded yes, barriers to increased physician FTE included: cost of hiring anesthesia doctors (*n* = 15, 58%), lack of support from the hospital (*n* = 14, 55%), lack of providers trained (*n* = 17, 77%), and lack of providers available (*n* = 20, 77%).

#### Services

28 institutions (82%) report having an acute pain program, of which it was more common for institutions to have physicians rather than PAs or other APPs in their practice. 18 programs (64%) report having 24/7 in-house coverage from the following: Pediatric Residents (2, 14%), Anesthesiology Residents (8, 57%), Anesthesiology Fellows (8, 57%), and APP (4, 29%). Four institutions did not provide information regarding 24/7 in-house coverage. 26 institutions (77%) report providing pain interventional treatments, with 9 (27%) reporting the use of invasive devices (i.e., intrathecal pumps, spinal cord stimulators, peripheral nerve stimulators) in their program. 24 programs (71%) report having a pediatric headache program at their institution which is housed under varying clinics (62% in pediatric neurology, 17% in pediatric pain, 17% in pediatric neurology and pain headache clinics, 4% neither).

#### Pediatric pain fellowship

10 programs (29%) report having an ongoing pediatric pain fellowship program. Six of these programs are tied to fellowships in pediatric anesthesiology, two were independently ACGME approved, and two related to their institution's adult pain fellowship program.

### Survey 2

65 US-based physicians completed the survey. 88% of respondents report board certification in pediatrics and 66% hold sub-certifications. The most common sub-certifications were in Pediatric Emergency Medicine (PEM) (*n* = 19), Hospice and Palliative Medicine (*n* = 10), and Pediatric Rheumatology (*n* = 7). 26% report spending 76%–100% of their time over the past 4 years in Pain Medicine, which includes clinical, research, education, and administration activities. 10% report spending 51%–75% of their time, 19% reported 25%–50% of their time, and 45% reported 0%–25% of their time in Pain Medicine. For those who responded regarding clinical activities within Pain Medicine (*n* = 61), average distribution of effort was as follows: inpatient acute pain management (32%) outpatient chronic pain management (28%), inpatient chronic pain management (6%), outpatient headache pain management (5%), interventional pain management (19%), and other forms of clinical pain management including hospice, palliative, and emergency department (ED) pain management (6%). For those engaged in interventional pain management (*n* = 47) the following procedures were performed: Botox (*n* = 7), facial nerve blocks (*n* = 17), trigger point injections (*n* = 7), joint injections (*n* = 10), peripheral nerve blocks (*n* = 21), epidural injections (*n* = 7), facet injections (*n* = 6), acupuncture (*n* = 5), and massage/musculoskeletal manipulation (*n* = 2). See Table 2 for a summary of results.

**Table 2:** Descriptive statistics of survey 2 results.

	# of Physicians	Percentage
Certifications	65	100%
Board certified in pediatrics	57	88%
Hold sub-certifications	43	66%
Time Spent in Pain Medicine	62	100%
0–25%	28	45%
25–50%	12	19%
50–75%	6	10%
75–100%	16	26%
Clinical Activity in Pain Medicine (average distribution of effort)	61	100%
Inpatient acute pain mgmt		32%
Outpatient chronic pain mgmt		28%
Interventional pain mgmt		19%
Inpatient chronic pain mgmt		6%
Outpatient headache pain mgmt		5%
Other		6%
Interventional pain mgmt procedures	47	100%
Peripheral nerve blocks	21	45%
Facial nerve blocks	17	36%
Joint injections	10	21%
Botox	7	15%
Epidural injections	7	15%
Trigger point injections	7	15%
Facet injections	6	13%
Acupuncture	5	11%
Massage/musculoskeletal manipulation	2	4%

Respondents also provided qualitative responses of reasons for becoming board certified and perceived advantages of board certification. Reported advantages of becoming board certified include receiving formal recognition, credentials, and validation for a specific expertise which would allow families to find appropriate providers as well as show value to the hospital to increase resources. Other advantages include receiving optimal training on up-to-date information, increasing knowledge and competency in pediatric pain medicine.

### Survey 3

212 US-based residents completed the survey. 53.8% report having a dedicated pediatric pain service at their hospital and 38.2% report having a Pain Medicine elective available, 23.5% of which express having an interest in this rotation.

#### Educational needs

Reported areas of need regarding education included knowledge of analgesics (52.1%), general pain management (50.0%), and interventional pain medicine (33.7%). For general pain management, chronic pain management (22.1%), outpatient pain management (21.1%), and non-pharmacological interventions (9.5%) are reported as highest needs. Within interventional management, highest needs include nerve blocks (39.0%) and trigger point injections (12.5%).

#### Pain medicine capability

Respondents were asked to self-assess their capability in practicing pediatric pain medicine on a scale of 0–10, with capability defined as ability/skill, knowledge level, and comfort. Highest average capability scores were for sickle cell disease (*M* = 6.50) and opioids in the inpatient setting (*M* = 6.27). Lowest were for chronic musculoskeletal pain in the outpatient setting (*M* = 4.05) and procedures (*M* = 1.44 for peripheral nerve blocks; *M* = 1.24 for trigger point injections). 52 respondents report having performed nerve blocks in the past, the most common of which were digital (*n* = 27), dorsal penile (*n* = 15), peripheral (*n* = 3), and sphenopalatine ganglion (*n* = 2).

#### Career interest

62 (29.2%) respondents report having interest in post residency Pain Medicine training, 12 of which (19.3%) being “very interested” or “extremely interested”. When asked what would interest them about a career in Pain Medicine, most reported interest in pain in youth (56.1%), to learn and perform procedures (47.6%), a positive work/life balance (40.1%), functioning as part of interdisciplinary teams (39.2%), to grow in a new field of study or care (33.0%), an academic interest in pain in children and young adults (21.1%), potential financial rewards (17.0%), and a personal experience with pain (8.0%). See Table 3 for a summary of results.

**Table 3:** Descriptive statistics of survey 3 results.

	# of Residents	Percentage
Residency	212	100%
Dedicated pediatric pain service	114	53.8%
Pain Medicine elective available	81	38.2%
Educational Needs	190	100%
Knowledge on analgesics	99	52.1%
General pain management	95	50.0%
*Chronic pain management*	21	22.1%
*Outpatient pain management*	20	21.1%
*Non-pharmacological interventions*	9	9.5%
Interventional pain medicine	64	33.7%
*Nerve blocks*	25	39.0%
*Trigger point injections*	8	12.5%
Interest in Fellowship	62	100%
Not at all interested	1	1.6%
Slightly interested	25	40.3%
Moderately Interested	24	38.7%
Very Interested	9	14.5%
Extremely Interested	3	4.8%
Reasons for Interest in Pain Medicine	212	100%
Clinical interest in pain in youth	119	56.1%
Learning and performing procedures	101	47.6%
Positive work/life balance	85	40.1%
Interdisciplinary teams	83	39.2%
Grow in a new field of study or care	70	33.0%
Academic interests in pain in youth	45	21.1%
Potential financial rewards	36	17.0%
Personal experiences with pain	17	8.0%

Respondents also provided qualitative responses on information needed in order to pursue a career in Pediatric Pain Medicine. The majority reported needing details on job prospects, work-life expectations, integration with other practices, scope of practice/day-to-day responsibilities, and career outlook. Other requested information surrounded knowing the length of fellowship and what it would specifically entail.

## Discussion

Pediatric Pain Medicine emerged as a distinct medical specialty in the late 20th century (12, 13). Before that, there were biases leading to the under-treatment of pain in children including that they were felt to not experience pain, or even if they did, they could not be safely managed with analgesics (14, 15). It was felt that untreated pain would have no long-term consequences. In the last 40 years, there has been tremendous growth in the field with the emergence of acute pain services focused on the unique needs of children (16) and multidisciplinary pain clinics to address chronic pain in youth utilizing a biopsychosocial approach (17, 18). Despite all of these advances, recent reports find that Pediatric Pain Medicine, among many other specialties, is experiencing a crucial workforce shortage which is associated with longer wait times and distances to travel for care (6, 7). There are currently limited opportunities to receive formal training in the field with eight pediatric-focused fellowship programs that are accredited for the American Board of Anesthesiology (11). The path to board certification, essential for growth and development of the specialty, is open only to those who have training in Anesthesiology, Physical Medicine and Rehabilitation, Family Medicine, Psychiatry and Neurology.

Our expert panel developed and distributed three surveys to examine resources in Pediatric Pain Medicine. The first examined the 47 pediatric comprehensive pain programs in the US at the time, the second investigated current practice patterns of US pediatricians practicing Pain Medicine as well as their opinions and motivations surrounding Pain Medicine board certification, and the third examined current pediatric residents’ exposure to and interest in Pediatric Pain Medicine. These surveys are a part of a broader effort on the part of our work group to develop solutions to Pediatric Pain Medicine workforce shortages and patient access challenges, potentially by developing pathways for pediatricians to obtain board certification in Pain Medicine.

Our surveys highlight some important points regarding present issues and future concerns for the field of Pediatric Pain Medicine. First, there are only 47 programs that were identified using presently available resources (9). While not specifically analyzed using our surveys, pediatric pain centers are concentrated in population centers, leaving large geographic areas of the US uncovered, with only about half of the states in the US having even one pain center (9). Second, in the available programs, according to our survey, wait times for an initial evaluation are two months or longer for two-thirds of centers. Given that initial referral to a pediatric pain program often occurs after several other evaluations and months of time, this is certainly too long to wait for a child with an untreated pain problem (8). There is scarce public data on the number of pain physicians per state and the average number of patients seen per center, which could be important to know and highlight the growing concern in this field. Third, as our first survey demonstrates, there are significant barriers to increasing staff size and resources including the cost of providers in the current model, lack of hospital support, and inadequate numbers of trained and or available physicians. According to the 2021–2022 academic year from the Association of American Medical Colleges Medical School Faculty Salary Survey report, anesthesiologist salaries range on average from $354,913 to $519,652, while pediatricians salaries range on average from $187,604 to $340,408 (19). Additionally, of the existing comprehensive pain programs in the US, 38 (66%) offer interventional procedures which may be administered by anesthesiologists, but could be delivered instead by Pediatricians certified in Pain Medicine. Therefore, it may be appropriate to train pediatricians in pain medicine in order to save costs, increase staff size, and improve institutional support.

There are a number of pediatricians who are already practicing Pain Medicine. Over half of those who answered our survey spend more than 25% of their time on pediatric pain activities. They are involved in a range of practices including acute, chronic, and interventional pain management. There was also enthusiasm from those surveyed for the potential to obtain board certification should it become available. Additionally, our third survey illustrates that there is interest among current residents in pursuing Pediatric Pain Medicine. 29.2% of the sample demonstrated some form of interest, but they also highlighted needing to know logistics surrounding a potential fellowship and what job prospects would look like, as well as how a pediatric pain fellowship program could provide better career opportunities compared to that of a general pediatrician. Additionally, many reported educational needs within a fellowship program in order to practice Pain Medicine, particularly surrounding analgesics, general pain management, and interventional procedures. Thus, it seems that future growth in the field might be generated from raising awareness, increasing general education about, and expanding training opportunities for pediatric-trained physicians in pain medicine.

This workforce study is not without limitations. Response rates for online research surveys are often suboptimal, with multiple factors identified from survey development to delivery stages (20). While 72% of the 47 identified comprehensive pediatric pain programs responded to the initial survey, some trends or practice patterns may have been missed. Additionally, without a centralized and comprehensive directory of pediatric pain clinics in the US, it is possible that programs were omitted that might have enhanced the data and added perspectives. We also did not collect data on independent functional abdominal pain clinics which we did with independent headache clinics. Similarly, in the second survey, only 65 responses were received in total from physicians practicing Pediatric Pain Medicine. Since we were unable to survey all pediatric pain physicians in the US, we cannot generalize their responses to the pediatric pain physician workforce as a whole. If anything, these results give an idea as to what time percentages spent in Pain Medicine, distributions of effort, and pain management procedures could be. Lastly, only 212 out of a possible approximately 10,000 pediatric residents responded to the third survey, potentially limiting the generalizability to this population. This response rate is disappointing, but study by Saleh and Bista regarding online surveys for research suggests that this is not entirely surprising (21). They found that respondents reported being more likely to complete a survey if they are pre-notified, that the survey is posted by trainees or people that they know, or if the survey is recommended by an authority figure in their training. This explains, in part, the low response rate from pediatric residents who did not had pre-notification or influence from their mentors or leaders. Future studies could address some of these methodologic issues, should the need for further pediatric resident survey data be required.

These findings outline the capacity and practice patterns of most pediatric comprehensive pain programs in the US as well as physician perspectives on barriers and opinions on receiving board certification. These results highlight the need for increased opportunities for training in pediatric pain medicine in order to support limited access to care due to workforce shortages (6, 7). Increasing the pain-trained workforce is key to overcoming one of the largest barriers to care (10). While some of the gap can be filled by increasing exposure to pain medicine in residency and training more nurse practitioners and physician assistants in pain medicine, the complexity of the field requires more fellowship trained physicians. There were respondents who are interested in pursuing formalized training and a career in Pediatric Pain Medicine, which is a promising sign for the future. Conceivably, greater exposure to pain management concepts in residency may promote the interest of pediatricians in pursuing further training. This could be the subject of future inquiry and potential action. There are currently only eight accredited programs through the ACGME that provide fellowship training in Pediatric Pain Medicine in the US (11), only two of which are independently accredited within pediatric medical centers. None of these fellowship training programs are available to Pediatricians, however, only Anesthesiologists, Physiatrists, Psychiatrists, and Neurologists. Additionally, pain management educational requirements through ACGME Pediatrics programs are specialty-dependent and do not cover Pediatric Pain Medicine as a field (22). Thus, it is paramount that a formal pathway to Pediatric Pain Medicine be developed as a fellowship program for Pediatricians to increase the number of trained physicians and address the pressing needs of this population. Unfortunately, despite the potential development of such a fellowship program, board certification in Pediatric Pain Medicine will not be possible without inclusion of Pediatricians by the ACGME. One might argue that a clinical training program alone without certification would be sufficient. However, without formal recognition, the path to full time practice and reimbursement as a specialist is unclear and thus hinders trainees from seeking such training. Results from these surveys highlighting the current state of US pediatric pain programs, the need of additional training opportunities, and interest among both those in practice and training should be presented to board representatives within Pediatrics to hopefully incorporate these changes within the ACGME.

## Data Availability

The original contributions presented in the study are included in the article/Supplementary Material, further inquiries can be directed to Christopher Theriault, ctheriault@connecticutchildrens.org.
